# Hsa_circRNA_001859 regulates pancreatic cancer progression and epithelial-mesenchymal transition through the miR-21-5p/SLC38A2 pathway

**DOI:** 10.3233/CBM-220229

**Published:** 2023-05-15

**Authors:** Liang Li, Nan Wang, Jun Wang, Jiangang Li

**Affiliations:** a Department of General Surgery, The Second Affiliated Hospital of Xinjiang Medical University, Urumqi, Xinjiang, China; b Department of Pancreatic Surgery, The First Affiliated Hospital of Xinjiang Medical University, Urumqi, Xinjiang, China

**Keywords:** Pancreatic cancer, circ_001859, miR-21-5p, SLC38A2

## Abstract

**OBJECTIVE:**

This study attempts to investigate whether hsa_circRNA_001859 (circ_001859) could regulate the proliferation and invasion of pancreatic cancer through the miR-21-5p/SLC38A2 pathway.

**METHODS:**

GSE79634 microarray was analyzed with R package. The expression of circ_001859 in pancreatic cancer tissues and cells was verified by qRT-PCR. After the overexpression of circ_001859, cell proliferation, cell migration and invasion were verified by colony formation and transwell assay. The targeting relationship between miR-21-5p and circ_001859 was predicted by TargetScan and was verified by dual luciferase reporter assay, RNA pull down and qRT-PCR. The effect of miR-21-5p on cell proliferation, migration and invasion were investigated by colony formation and transwell assay respectively. Similarly, the targeting relationship between miR-21-5p and SLC38A2 was predicted by TargetScan and was verified by dual luciferase reporter assay, western blot and qRT-PCR. The effect of SLC38A2 on cell proliferation was investigated by colony formation.

**RESULTS:**

Circ_001859 was lowly expressed in pancreatic cancer tissues and cells. *In vitro* assays showed that overexpression of circ_001859 could inhibit the proliferation, migration and invasion of pancreatic cancer. In addition, this effect was also confirmed in xenograft transplantation model. Circ_001859 could be bind to miR-21-5p and sponge its expression in pancreatic cancer cells. Overexpression of miR-21-5p enhanced the proliferation, migration and invasion ability of pancreatic cancer cells, while the inhibition of miR-21-5p expression suppressed these abilities. Moreover, miR-21-5p directly targeted at SLC38A2 and inhibited SLC38A2 expression levels while circ_001859 up-regulated SLC38A2 levels. SLC38A2 expression knockdown enhanced cell proliferation but SLC38A2 overexpression resulted in decreased proliferation, and effects of SLC38A2 could be rescued by miR-21-5p and circ_001859. In addition, both QRT-PCR and immunofluorescence confirmed that circ_001859 could regulate tumor epithelial-mesenchymal transition (EMT) through the miR-21-5p/SLC38A2 pathway.

**CONCLUSIONS:**

This study suggests that circ_001859 may inhibit the proliferation, invasion and EMT of pancreatic cancer through the miR-21-5p/SLC38A2 pathway.

## 1. Introduction

Pancreatic cancer is a leading cause of cancer death worldwide and its global burden has more than doubled over the past 25 years. The highest incidence regions for pancreatic cancer include North America, Europe and Australia, and although much of this increase is due to ageing worldwide populations, there are key modifiable risk factors for pancreatic cancer such as cigarette smoking, obesity, diabetes and alcohol intake. The prevalence of these risk factors is increasing in many global regions, resulting in increasing age-adjusted incidence rates for pancreatic cancer [1, 2]. Although great progress in diagnosis and treatment has been made, the 5-year survival rate remains dismal in pancreatic cancer [3]. Pancreatic cancer will probably move to the second primary cause of cancer death around the world by 2020 in the absence of improvements in treatment [2, 4]. Currently, there is no way for the effective diagnosis of early stages of pancreatic cancer [4]. Under this grim situation, it is imperative to understand its molecular mechanisms connected with initiation, progression, and therapy obstacle [5]. In spite of present inspection into some well-known genes, the treatment of pancreatic cancer is still limited. Thus, this study focused on new molecular mechanism to solve treatment obstacle.

Circular RNAs (circRNAs), a novel kind of widespread and diverse endogenous RNAs, have a significant effect on the expression of gene at post-transcriptional level and are extensively expressed in body cells [6]. Currently, circRNAs have been found to be widely expressed in the cytoplasm with the advent of RNA sequencing technology and their complexity of regulation, specificity of expression, and vital function drew concerns in various diseases [7]. CircRNA_100782 promoted pancreatic carcinoma by sponging miR-124 through the IL6-STAT3 pathway [8]. Besides, circ_0000977 silencing suppressed pancreatic cancer cell proliferation and induced cell cycle arrest which was simulated by increased miR-874-3p expression and decreased PLK1 expression [9]. Both results suggested circRNA could be significant in pancreatic cancer diagnosis and treatment.

MicroRNAs (miRNAs), short noncoding RNAs 20–24 nucleotides in length, regulate the gene expression after transcription. Importantly, miRNAs play critical roles in a broad range of biological proceedings, containing proliferation, differentiation, apoptosis, and stress response, linking them with numerous human diseases [10]. In recent years, accumulating evidence has also indicated that the dysregulation of miRNAs in various human cancers may modulate tumor cell proliferation, tumor angiogenesis, invasion and metastasis during tumor initiation and progression [11]. Several researches have suggested that miR-21-5p may play an important part in different diseases, including breast cancer, colorectal cancer and lung cancer [12, 13, 14]. No researches have discussed the intriguing role of miR-21-5p in pancreatic cancer and this study for the first time studied this point.

The transport of glutamine across the plasma membrane is regulated by two systems: the system ASC transporter SLC1A5 and the system A transporter SLC38A1, SLC38A2 and SLC38A4. SLC1A5 is a two-way transporter that transports Ala, Ser, Cys, Asn and Gln in an exchange manner. MYC oncogene can induce the expression of SLC1A5, and it has been found that the high expression of SLC1A5 is related to the poor prognosis of many tumors [15, 16]. It is reported that other glutamine transporters, such as SLC38A1, SLC38A2 and SLC7A5, also play an important role in tumors. Other studies have shown that SLC38A2 plays an important role in pancreatic cancer [17]. These data showed that the tumor cells lacking SLC38A2 fail to concentrate intracellular alanine and undergo a profound metabolic crisis resulting in markedly impaired tumor growth [17]. but the role of miR-21-5p and SLC38A2 in pancreatic cancer is still unclear. However, whether the expression of SLC38A2 is regulated by miRNA or its relationship with miR-21-5p has not been found.

This study intends to make a thorough inquiry about the molecule and biological signaling pathway in pancreatic cancer. Both *in vitro* and *in vivo* assays were applied to verify the prediction of function of circ_001859. Besides, the regulation mechanism via circ_001859-miR-21-5p-SLC38A2 axis was analyzed. This study might give a new sight into molecular mechanisms in pancreatic cancer treatment.

## 2. Experimental methods

### 2.1 Tissue samples

Twenty samples of pancreatic carcinoma tissues were obtained from patients with pancreatic cancer treated at The Second Affiliated Hospital Affiliated to Xinjiang Medical University. A total of 12 males and 8 females were included in this study. The average age was (57.91 ± 5.36) years, ranging from 37 to 68 years. According to the American Joint Committee on Cancer (AJCC) and the International Union against Cancer (UICC) criteria for TNM staging of pancreatic cancer, there were 3 cases of stage I, 7 cases of stage II, 6 cases of stage III, and 4 cases of stage IV. None of the patients with pancreatic cancer underwent preoperative radiotherapy or other cancer-specific therapies. Pancreatic tissues removed freshly were immediately frozen in liquid nitrogen and stored at -80${}^{\circ }$C until RNA extraction. This study protocol had been approved by the Clinical Research Ethics Committee of the Second Affiliated Hospital of Xinjiang Medical University.

### 2.2 Microarray analysis

In the GEO database, GSE79634 was analyzed by chip using the GPL19978 platform (Agilent-069978 Arraystar Human CircRNA microarray) [18]. circRNA expression levels in 20 pancreatic ductal adenocarcinoma samples and 20 normal pancreatic cancer adjacent tissues were analyzed and distinguishable circRNAs were identified. The pathologic validation of all tumor tissues was confirmed by the research group of Wang et al. [18]. Differentially expressed circRNAs were chosen with Fold change values > 2 and P< 0.05 as screening conditions, and volcanic maps and heat maps of differentially expressed genes were drawn using R language.

### 2.3 Cell culture

Pancreatic cancer cell line Panc-1, Capan-1, and normal ductal epithelial cell line HPDE6-C7 were acquired from the American Type Culture Collection (ATCC, Manassas, VA, USA) and maintained according to ATCC’s cell media recommendations in standard conditions (37${}^{\circ }$C, 5% CO${}_{2}$). Media was Dulbecco’s modified Eagle medium (DMEM, Grand Island, NY, USA) supplemented with 10% fetal bovine serum (FBS, South Logan, UT, USA). HEK293 cells for transfection were also bought from ATCC and cultured in DMEM supplemented with 5% FBS.

### 2.4 Fluorescence in situ hybridization (FISH)

For FISH assay, Fluor 594-labeled probe for detecting hsa_circRNA_001859 and Alexa Fluor 488-labeled probes for detecting miR-21-5p were synthesized by GenePharma. After fixation, cells were incubated with pre-hybridization buffer, then, hybridization was conducted at 55${}^{\circ }$C for 2 h. Afterwards, the nuclei were stained with DAPI. The probe signals were determined with the FISH Kit (RiboBio, Guangzhou, China) based on the manufacturer’s instructions. The sequence of hsa_circRNA_001859 FISH was: 5${}^{\prime }$- ACCGAGGAGGAGATCTGACTGCA-3${}^{\prime }$.

### 2.5 RT-PCR

Total RNA of samples was extracted using TRIzol${}^{\text{®}}$ reagent (Invitrogen, Carlsbad, CA, USA). CircRNAs were amplified by divergent primers and all primer sequences were listed in Table S1. After quantification with NanoDrop 2000 (Thermo Fisier Scientific Inc, USA), total RNA was reverse transcribed using Prime Script RT reagent Kit (TaKaRa, Tokyo, Japan) and SYBR Premix Ex Taq II (TaKaRa). Three replicates were executed in every group according to the contents of internal reference U6 and GADPH, and 2${}^{-\Delta \,\Delta \,\text{CT}}$ method was adopted to calculate relative expressions.

### 2.6 Western blot

Total protein solution was obtained by adding RAPI protein buffer (Beyotime, Shanghai, China). And the protein concentration was measured by Pierce BCA Protein Assay Kit (Pierce, Rockford, IL, USA). After denaturation, the protein received SDS-PAGE electrophoresis and was transferred to a PVDF membrane by using 200mA constant current for about 120 min. The membrane was blocked and incubated with primary antibodies (anti-SLC38A2 ab157492, 1:1000; anti-GAPDH, ab181603, 1:10000, Abcam, Cambridge, MA, USA) at 4${}^{\circ }$C overnight and secondary antibodies for 2 h at room temperature. The membrane was colorized using Life Technology’s ECL Plus. According to analysis system software and Lab Works 4.5 image acquisition, the integral optical density of the protein bands was identified.

### 2.7 Cell transfection

PcDNA3.1/circ_001859 (pcDNA3.1 as control), pcDNA3.1/SLC38A2 (pcDNA3.1 as control), miR-21-5p-mimics and miR-21-5p-inhibitor were purchased from Shanghai Biotech, Shanghai, China. Interfering nucleotide sequence was designed according to Invitrogen RNA interference sequence design software (BLOCK-iTTM RNAi Designer). At the same time, a negative control was designed according to an unrelated nucleic acid sequence which has the same base number. PANC-1 and Capan-1 cells were seeded in 6-well plates and cultured to 60–70% confluence before transfection, negative controls and overexpression vectors were transiently transfected using Lipofectamine 2000 (Invitrogen) according to instructions and then incubated in a 5% CO${}_{2}$ atmosphere for 48 h at 37${}^{\circ }$C.

### 2.8 Colony formation

Transfected cells were seeded into a 6-well plate at a density of 1 × 10${}^{3}$ cells/well and shaken for well disperse. Then they were placed into the constant temperature chamber in an atmosphere of 5% CO${}_{2}$ at 37${}^{\circ }$C for 2 weeks incubation. When colonies were visible (> 200 cells per colony), the incubationwas ended. Cells were then washed by PBS and fixed by 4% paraformaldehyde for 15 min. After removing the stationary liquid, cells were stained with crystal violet for 10–30 min. Next, the dye liquor was discarded and cells were subsequently dried. Five random fields of view were selected and cell numbers were counted.

### 2.9 Transwell migration and invasion experiment

Transwell assay was carried out using 24-well transwell inserts (Corning, Corning, NY, USA) according to the manufacturer’s protocol. Transfected cells were collected, resuspended in serum-free medium and transferred to permeable pores. Cells were seeded at a density of 3 × 10${}^{5}$ per insert and the lower chamber of the Transwell was filled with 500 μL DMEM supplemented with 10% FBS. The chambers were then incubated for 24 h in culture medium with 10% FBS in the bottom chambers before examination. The cells on the upper surface were scraped and washed away, whereas the migrated cells on the lower surface were fixed and stained with 0.05% crystal violet for 30 min. Finally, 5 independent fields were counted for average number of cells. For cell invasion, were seeded in the matrigel-coated transwell insert. The cells were then processed similar to that of cell migration assay. Cells that had invaded through the Matrigel to the bottom of the insert were fixed, stained, photographed and quantified in 5 random high-powered fields.

### 2.10 Dual luciferase reporter assay

The circ_001859-wt, circ_001859-mut, SLC38A2-wt, and SLC38A2-mut obtained from Shanghai Biosciences were cloned into the pmirGLO plasmid, the empty plasmid, pmirGLO-NC, pmirGLO-circ001859, miR-21-5p mimics, miR-21-5p inhibitor were cotransfected into HEK293 with Lipofectamie 2000 (Invitrogen, Carlsbad, CA, USA). Luciferase activity was detected on a microplate reader using the dual luciferase reporter assay kit (Promega, Madison, WI, USA) and the experiments were repeated for three times.

### 2.11 RNA pull down experiment

Circ_001859-wt and control group probe were in vitro transcribed and biotin-labeled by using the biotin RNA labeling mix (Roche, Basel, Switzerland), treated with RNase-free DNase I (Roche) and purified using an RNeasy mini kit (Qiagen, Valencia, CA, USA). Then cell protein (1 mg) extract was mixed with biotinylated RNA biotin-labeled RNAs (50 pmol), incubated with streptavidin agarose beads (Invitrogen), and washed three times with NaCl/Pi at room temperature. Dynabeads were used for western blotting. After being washed, mixed with cell lysate, 30 μl of cold 0.1% SDS buffer was added to beads and heated at 95${}^{\circ }$C for 2 minutes. After the sample was centrifuged, the supernatant was taken for RNA extraction and qPCR experiments.

### 2.12 Xenograft transplantation model

BALB/c nude mice were purchased from Charles River (Beijing, China). These mice were given free access to sterile food and water during the whole experiment proceedings. All animal experiments with nude mice were performed strictly in accordance with a protocol approved by The Second Affiliated Hospital of Xinjiang Medical University. Transfected cells were made into suspension in each group (2 × 10${}^{6}$ cells/ml) andinjected subcutaneously into nude mice, 4 nude mice in each group. u Tumor volume was. The tumor volume of mice was measured electronically three times a day and calculated according to the volume V= length × width × width/2. The tumor weight of mice was measured three times on an electronic balance. After 6 weeks, the nude mice were killed and the tumorigenesis was observed.

### 2.13 Hematoxylin and eosin (HE) staining

HE staining was used to detect the overall condition of tumor tissue. In this study, hematoxylin and eosin was used to stain paraffin sections of mouse tumor tissues after deparaffinization and dehydration. Finally, observe the cell morphology under a microscope.

### 2.14 Immunohistochemistry assay (IHC)

KI67 staining was used to detect cell proliferation in tumor tissues. Paraffin sections of xenograft tumor tissues were cut with a thickness of 4 μm. Sections were incubated with primary antibodies: anti-Ki67 (Abcam, #ab15580, 1 μg/ml). The temperature was maintained at 4${}^{\circ }$C overnight. Sections were co-incubated with HRP-polymer-conjugated secondary antibodies after washing with phosphate-buffered saline, then, they were immunostained by using DAB plus kit.

### 2.15 Immunofluorescence (IF)

TUNEL and EMT-related proteins were stained by using fluorescent staining to verify tumor tissue apoptosis and EMT progression. Paraffin sections of xenograft tumor tissues were cut with a thickness of 4 μm. Sections were incubated with primary antibodies: anti-E-cadherin (Abcam, # ab40772, 1:500) and Vimentin (Abcam, # ab92547, 2 μg/ml). The temperature was maintained at 4${}^{\circ }$C overnight. The slides were washed in PBS for 3 times, 5 min each time. After the sections were slightly dried, the secondary antibody of the corresponding species of the primary antibody was added and incubated for 50min at room temperature and away from light. Sections were washed 3 times with PBS, 5 min each time. After PBS was removed, DAPI dye was added and incubated for 10 min at room temperature away from light. The sections were observed under fluorescence microscope and the images were collected.

### 2.16 Statistical analysis

Shapiro-Wilk normality test was used to verify the distribution of data in this study before the analysis began. Data conforming to normal distribution was represented as mean ± standard deviation (Mean ± SD). Two-tailed t-test was used for comparison between groups. One-way ANOVA was used for comparison of several groups. Statistical analysis was performed using GraphPad Prism 6.0 software (La Jolla, CA, USA).

## 3. Results

### 3.1 Circ_001859 in was lowly expressed in pancreatic cancer tissues and cells

**Figure 1.**
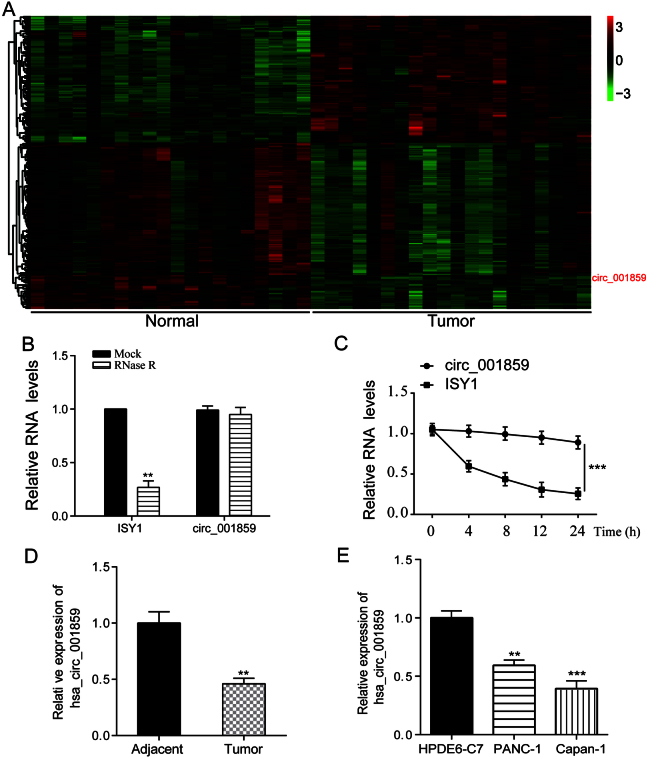
Circ_001859 was down-regulated in tumor tissues and cells. (A) GSE79634 revealed circ_001859 as a suppressed circRNA with a criterion of logFC > 2 and p< 0.05. (B) The expression of circ_001859 and ISY1 after RNase R treatment was detected by QRT-PCR. (C) The degradation of circ_001859 was verified by half-life detection. (D) Circ_001859 was lowly expressed in tumor tisseus. (E) Circ_001859 was lowly expressed in pancreatic cancer cell lines PANC-1 and Capan-1. P**< 0.01, P*⁣**< 0.001 indicated statistical significance.

The GSE79634 microarray was selected from the GEO database for analysis. Differentially expressed circRNAs in pancreatic cancer and normal control specimens were analysed by R software, and the heat map was plotted. LogFC > 2 and p< 0.05 were used as the criterion for differentially expressed circRNAs. The results show that hsa_circ_001859 was lowly expressed in pancreatic cancer (Fig. 1A). As far as we know, this is a newly discovered circRNA, so firstly, we found that RNase R can effectively degrade the expression of its parent gene ISY1 but cannot degrade the expression of circ_001859 after treatment with RNase R (Fig. 1B, P< 0.01). Further half-life tests also found that circ_001859 would not be degraded. These data demonstrate the cyclic properties of circ_001859 (Fig. 1C, P< 0.001). Total RNA was collected from 20 pairs of tumor and normal tissues and expression level of circ_001859 was detected by qRT-PCR assay. The results showed that the expression level of circ_001859 in tumor tissues were remarkably down-regulated compared with normal tissues (Fig. 1D, P< 0.01). Similarly in pancreatic cancer cells, the expression level of circ_001859 in PANC-1 and Capan-1 was lower compared with normal HPDE6-C7 cells (Fig. 1E, P< 0.01, P< 0.001).

### 3.2 Circ_001859 overexpression suppressed proliferation, migration and invasion of pancreatic cancer cells

**Figure 2.**
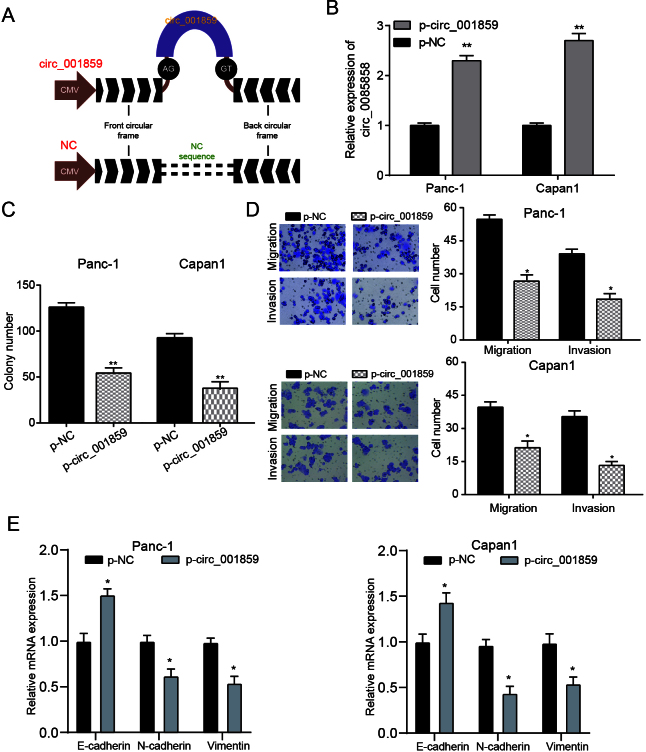
Circ-001859 suppressed cell proliferation, migration and invasion. (A) Sketch of structures of p-circ-001859 and NC vector were shown (B) Circ_001859 expression was enhanced in p-circ_001859 group. (C) Circ_001859 inhibited cell proliferation according to colony formation experiment. (D) Transwell experiment also verified that the overexpression of circ_001859 suppressed invasion and migration (E) The expression levels of EMT markers (E-cadherin, N-cadherin and Vimentin) were detected by QRT-PCR. P**< 0.001, P*< 0.05 indicated statistical significance.

We constructed a circ_001859 overexpression plasmid (p-circ_001859) with circular frame and circ_ 001859 sequence (Fig. 2A) and qPCR verified that circ_001859 was significantly overexpressed in PANC-1 and Capan-1 cells in p-circ_001859 group (Fig. 2B, P< 0.01). Overexpression of circ_001859 suppressed cell proliferation (Fig. 2C, P< 0.01). Transwell experiments also confirmed that overexpression of circ_001859 significantly inhibited cell invasion and migration (Fig. 2D, P<0.05). Further, we detected the markers of epithelial-mesenchymal transition (EMT) and found that circ_001859 significantly inhibited the EMT proceedings (Fig. 2E, P< 0.05).

### 3.3 Circ_001859 overexpression suppressed tumor growth in pancreatic mice model

**Figure 3.**
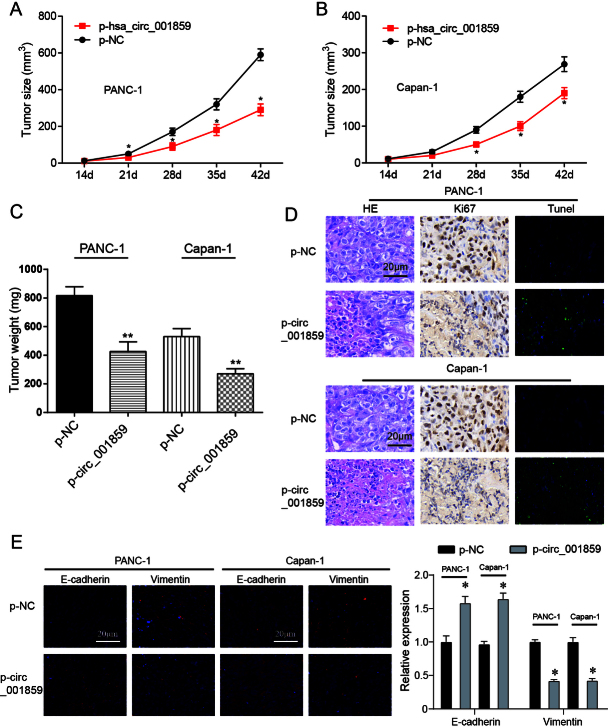
Circ_001859 overexpression suppressed tumor growth. (A–B) The tumor volume of mice in p-circ-001859 group was smaller. (C–D) Images and weight of tumors in nude mice indicated that circ-001859 overexpression inhibited tumor weight growth. (E) HE, KI67 and immunofluorescence detected the regulatory effect of circ_001859 overexpression on tumor proliferation, apoptosis and EMT process (× 200). P*< 0.05, P**< 0.01 indicated statistical significance.

Tumor volume of mice was measured every three days. It was discovered that circ_001859 overexpression significantly suppressed tumor volume growth (Fig. 3A–B, P< 0.05). Comparing the tumor size, tumors in p-circ_001859 group were smaller than those in p-NC group (Fig. 3C, P< 0.01). Similarly, tumors in p-circ_001859 group weighted less compared with p-circ_008585 group (Fig. 3D, P< 0.01). Finally, we used HE, KI67 and immunofluorescence to verify tumor proliferation, apoptosis and EMT markers and found that circ_001859 could promote cell proliferation and inhibit apoptosis. In addition, overexpression of circ_001859 can significantly inhibit the EMT proceedings, which is similar to our in vitro experimental results (Fig. 3E, P< 0.05). This experiment therefore confirmed that the overexpression of circ_001859 could suppress tumor growth in pancreatic cancer. 

### 3.4 Direct interaction between circ_001859 and miR-21-5p

**Figure 4.**
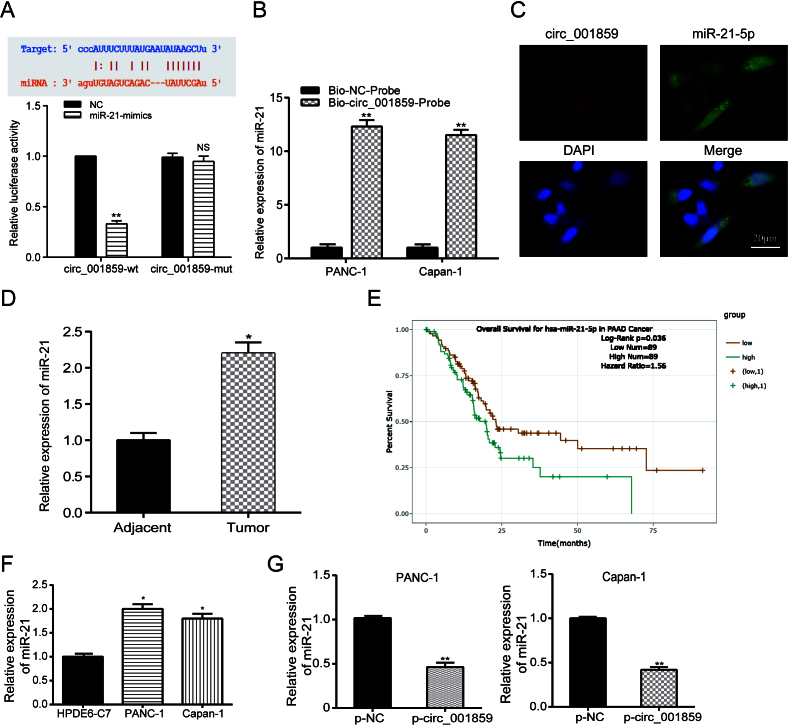
MiR-21-5p was up-regulated in cancer and could be suppressed by circ_001859. (A) Possible sequence of binding sites in the prediction between miR-21-5p and circ_001859. The luciferase reporter assay indicated that miR-21-5p-mimics directly targeted at circ_001859. (B) Circ_001859 pulled down miR-21-5p expression by RNA pull down assay. (C) The FISH assay verified the expression and localization of circ_001859 and miR-21-5p in pancreatic cancer cells (x 200). (D) MiR-21-5p expression was enhanced in tumor tissues and cells. (E) TCGA data validated the overall survival of miR-21-5p in pancreatic cancer. (F) MiR-21-5p expression was enhanced in cells. (G) p-circ_001859 suppressed miR-21-5p expression. P**< 0.01 indicated statistical significance.

The Starbase database was used to predicted the direct targeting relationship between miR-21-5p and circ_001859. In dual luciferase reporter assay, it can be founded that the fluorescence intensity in circ_001859-wt+miR-21-5p mimics group was remarkably lower compared with NC group. However, the fluorescence intensity in miR-21-5p mimics+circ_00858558 did not change significantly (Fig. 4A, P< 0.01). Next, we further demonstrated that biotinylated circ_001859 could pull down miR-21-5p. (Fig. 4B, P< 0.01). Further, we used FISH experiment to co-localize circ_001859 and miR-21-5p and found that the two were mainly expressed in the cytoplasm and co-localized in cells (Fig. 4C). These results suggests that they are able to bind to each other and function. In tumor tissues, miR-21-5p was found to be overexpressed in tumor tissues (Fig. 4D, P< 0.05). At the same time, we found that high expression of miR-21-5p in the TCGA database was associated with poor prognosis in pancreatic cancer (Fig. 4E, P< 0.05). In cancer cells as well, miR-21-5p showed greater expression in PANC-1 and Capan-1 cells than in human normal pancreatic ductal epithelial cell line HPDE6-C7 (Fig. 4F, P< 0.05). QRT-PCR indicated that miR-21-5p in p-circ_001859 group was down-regulated remarkably after circ_001859 overexpression compared with p-NC group (Fig. 4G, P< 0.0001).

### 3.5 MiR-21-5p mimics promoted proliferation, migration and invasion

**Figure 5.**
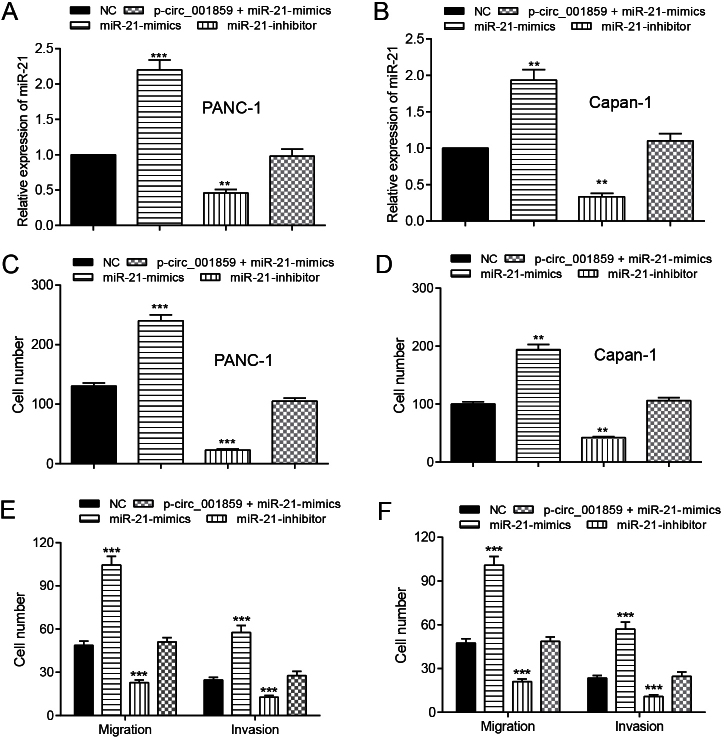
MiR-21-5p mimics promoted cell proferation, migration and invasion. (A–B) The expression of miR-21-5p in miR-21-5p-mimics, miR-21-5p-inhibitor, p-circ_001859+miR-21-5p-mimics in PANC-1 and Capan-1 cell lines was measured by qRT-PCR. (C–D) miR-21-5p-mimics promoted cell proliferation and miR-21-5p-inhibitor suppressed proliferation. (E–F) Transwell indicated that miR-21-5p mimics promoted cell migration and invasion. P*⁣**< 0.001, P**< 0.01 indicated statistical significance.

In order to study the regulation of miR-21-5p on the ability of invasion, proliferation and migration, four groups were divided as empty vector NC, miR-21-5p-mimics, miR-21-5p-inhibitor and p-circ_001859 + miR-21-5p-mimics group. The expression of miR-21-5p was significantly upregulated in miR-21-5p-mimics group and significantly downregulated in miR-21-5p-inhibitor group according to qRT-PCR, while miR-21-5p expression level in p-circ_001859 + miR-21-5p-mimics group was not quite different from the control group (Fig. 5A–B, P< 0.001, P< 0.01). Then we measured the ability of proliferation, migration and invasion of cells according to colony formation experiments and transwell experiments. The results verified that miR-21-5p-mimics promoted while miR-21-5p-inhibitor inhibited cell proliferation compared with the NC group. p-circ_001859 + miR-21-5p-mimics caused no significant changes in cell proliferation (Fig. 5C–D, P< 0.001). MiR-21-5p-mimics promoted while miR-21-5p-inhibitor suppressed cell migration and invasion but p-circ_001859 + miR-21-5p-mimics imposed no obvious influences on cell migration and invasion (Fig. 5E–F, P< 0.001). The above tests testified that miR-21-5p mimics promoted abilities of invasion, proliferation and migration of pancreatic carcinoma cells but the overexpression of circ_001859 restored the tumorigenicity of miR-21-5p mimics.

### 3.6 SLC38A2 was regulated by miR-21-5p and circ_001859

**Figure 6.**
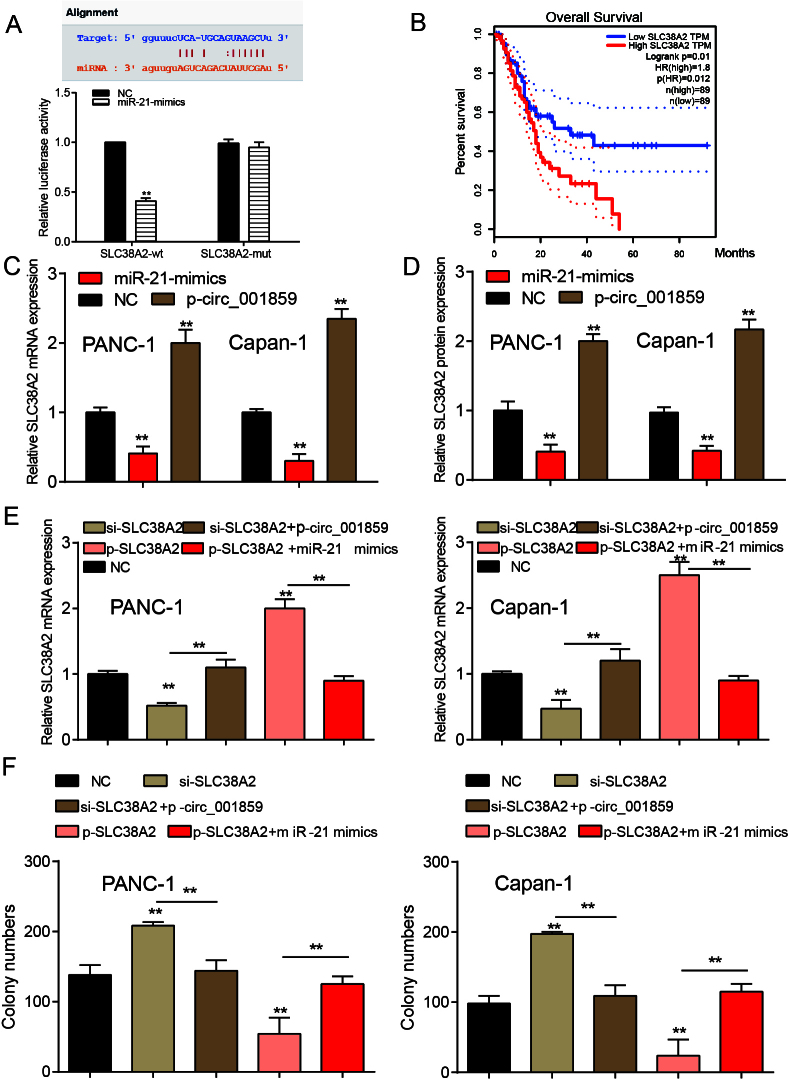
MiR-21-5p suppressed SLC38A2 expression and SLC38A2 suppression promoted cell proliferation. (A) MiR-21-5p directly targeted at SLC38A2. (B) TCGA data validated the overall survival of SLC38A2 in pancreatic cancer. (C–D) MiR-21-5p mimics suppressed SLC38A2 mRNA or protein expression but p-circ_001859 enhanced SLC38A2 expression. (E) Si-SLC38A2 suppressed SLC38A2 protein and mRNA expression and p-circ_001859 could restore the suppression. p-SLC38A2 enhanced SLC38A2 protein and mRNA expression and miR-21-5p mimics rescued the enhancement. (F) Si-SLC38A2 increased cell proliferation and p-SLC38A2 suppressed the proliferation. P**< 0.01, P*⁣**< 0.001 indicated statistical significance.

**Figure 7.**
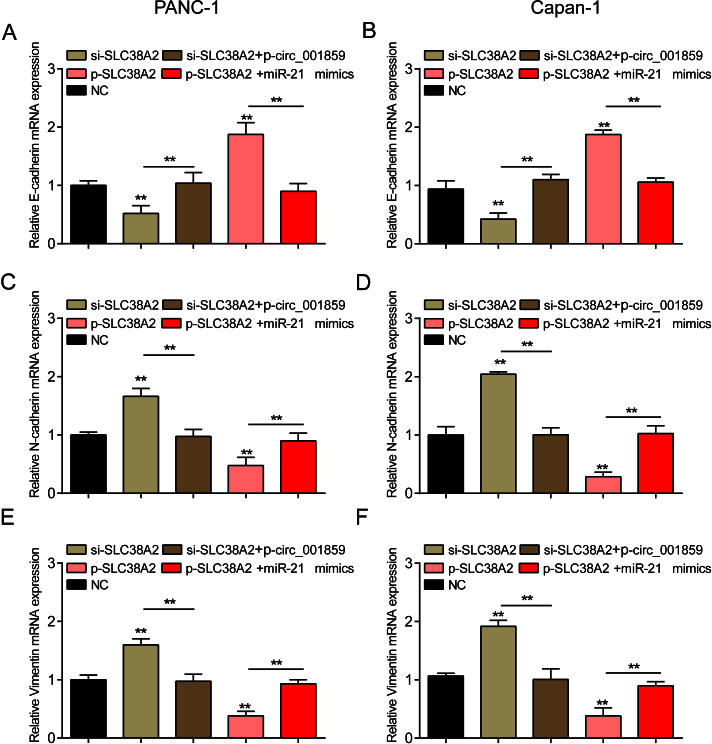
Circ_001859 affects EMT process through miR-21-5p/SLC38A2. (A–B) The expression level of E-cadherin mRNA was detected by QRT-PCR in each treatment group. (C–D) The expression level of N-cadherin mRNA was detected by QRT-PCR in each treatment group. (E–F) The expression level of Vimentin mRNA was detected by QRT-PCR in each treatment group. P**< 0.01, indicated statistical significance.

By bioinformatics analysis, we predicted the protein SLC38A2 that may be regulated by miR-21-5p. The dual luciferase assay showed that the SLC38A2-wt group’s fluorescence intensity decreased remarkably, however, the fluorescence intensity of miR-21-5p-mimics or NC group remained the same (Fig. 6A, P< 0.01). In addition, we used the TCGA database to analyze the relationship between SLC38A2 and pancreatic cancer prognosis and found that high expression of SLC38A2 was significantly correlated with poor prognosis (Fig. 6B, *HR*= 1.8, P= 0.01). Further, we verified the expression of miR-21-5p and circ_001859 on SLC38A2 mRNA and protein and found that miR-21-5p mimics could inhibit the expression of SLC38A2 (Fig. 6C–D, P< 0.01). However, circ_001859 overexpression could promote the expression of SLC38A2 (Fig. 6C–D, P< 0.01). In addition, in order to verify the regulatory effects between circ_001859, miR-21-5p and SLC38A2, we constructed si-SLC38A2, p-SLC38A2, respectively. si-SLC38A2+p-circ_001859 and p-SLC38A2+miR-21 mimics group. First, we verified that their expression method of SLC38A2 was equivalent to that of reversing the expression of SLC38A2 when the intervention of miR-21-5p or circ_001859 was applied simultaneously (Fig. 6E, P< 0.01). This result further confirms the regulatory role of the three.

Finally, in order to verify the regulation of the three factors on the process of tumor cells, we used cloning and EMT marker detection respectively and found that overexpression of SLC38A2 or inhibition of SLC38A2 could reverse the regulatory effects of miR-21-5p or circ_001859 overexpression on the process of pancreatic cancer (Figs 6F and 7, P< 0.01). These data suggest that circ_001859 may regulate the proliferation and metastasis of pancreatic cancer through the miR-21-5p/SLC38A2 pathway.

## 4. Discussion

Pancreatic cancer is a lethal malignancy that responsible for about 227,000 deaths every year on a global scale [19]. There’s a grim five-year low survival rate of this disease, which is only 6%. Therefore, better treatment strategies are sorely needed and early detection is of critical importance [20]. In this study, the low expression of hsa_circ_001859 in tumor tissues was found after screening GEO data. qRT-PCR, RNA half-life assay and other methods to verify its expression and ring-like characteristics. *In vitro* and *in vivo* evidence suggests that overexpression of circ_001859 could inhibit the progression of pancreatic cancer. This is the first report of this molecule as a tumor suppressor gene in tumors. Further, through bioinformatics and molecular biological verification, we found that circ_001859 might regulate the proliferation, metastasis and EMT proceedings of pancreatic cancer through the miR-21-5p/SLC38A2 pathway. In addition, through our study of prognostic data, we believe that our data will increase the feasibility of this molecule in the selection of diagnostic markers for pancreatic cancer and the development of targets for targeted therapy.

This study indicated that circRNA circ_001859 was down-regulated in pancreatic cancer patients. Furthermore, the proliferation, migration and invasion ability of pancreatic cancer cells were inhibited by the overexpression of circ_001859. Nair et al. observed that a lot of circRNAs in normal-adjacent samples of ER+ subtype is negatively correlated to the proliferation score, so they concluded that circRNA frequency may have an significant effect on the breast cancer acting as a biomarker [22]. Moreover, Yang et al. suggested that circ-LDLRAD3 might become a biomarker in the process of pancreatic cancer [23]. Microarray assay was notably used for prediction of differentially expressed circRNA, and qRT-PCR was used for verification of discovered difference.

Besides, this study firstly demonstrated that miR-21-5p was overexpressed in pancreatic cancer tissues. Overexpression of miR-21-5p stimulated the proliferation, invasion and migration ability of pancreatic cancer cells. Recent literatures were referred to that Pai et al. have discovered that the numerous miRNAs were aberrantly expressed in pancreatic cancer. A number of them could prove to be effective drug targets; however, effective targeting of the affected organ and minimizing off-target effects of the miRNA-based therapeutic agent represent formidable challenges [20]. Meanwhile, it is now becoming more and more evident that miRNAs have the potential to be utilized as bio-markers for cancer diagnosis. For example, certain miRNAs such as miR-376a, miR-221, miR-155, miR-21-5p, miR-301, and miR-222 are overexpressed in pancreatic cancer [5]. This study then properly proved the oncogenic role of miR-21-5p via a regulation loop.

Finally, we investigated the relationship between miR-21-5p and SLC38A2, results demonstrated that SLC38A2 was the target protein of miR-21-5p, and inhibited by miR-21-5p, circ_001859 can affect SLC38A2 by regulating miR-21-5p. Several downstream proteins have been discussed to be tumor regulator in pancreatic cancer, such as FBXO11 [24], IL-6 [25] and RBM3 [26] except SLC38A2. The blank intersection was discussed in this report and SLC38A2 was supported to be a tumor suppressor in pancreatic cancer as well.

The role of circ_0001859 in cancer progression has not been fully figured out. The interactions between circRNAs and miRNAs on the process of pancreatic cancer remain largely confused [5]. MiR-21-5p, as one of miRNA, has been studied in pancreatic cancer rarely. However, in our study, we found the effect of SLC38A2 as a target protein of miR-21-5p. Nevertheless, in this research, there were still some defects which should be taken into account. For instance, the SLC38A2 was not the only one gene of miR-21-5p, but this study merely investigated SLC38A2. Thus, we set out to make some improvements in the subsequent work.

## 5. Conclusion

Taken all together, circ_001859 which was lowly expressed in pancreatic carcinoma could suppress the ability of proliferation, migration and invasion of pancreatic cancer cells by down-regulating miR-21-5pand up-regulating SLC38A2.

## Authors’ contributions

Conception: Li Jiangang.

Interpretation or analysis of data: Li Liang, Wang Nan, Wang Jun.

Preparation of the manuscript: Li Liang.

Revision for important intellectual content: Li Liang, Li Jiangang.

Supervision: Li Jiangang.

## Supplementary data

The supplementary files are available to download from http://dx.doi.org/10.3233/CBM-220229.

## Supplementary Material

Table S1 PCR Primer
