# Supplementary material for: Hsa_circRNA_001859 regulates pancreatic cancer progression and epithelial-mesenchymal transition through the miR-21-5p/SLC38A2 pathway
Source: Cancer Biomark. 2023 May 15;37(1):39–52. doi: 10.3233/CBM-220229 (PMC10200212; doi:10.3233/CBM-220229)
Supplement: Table S1 PCR Primer [file cbm-37-cbm220229-s001.docx]

Table S1 PCR Primer

| **Gene** | **Primer（5’-3’）** |
| --- | --- |
| **circ_001859** |  |
| Forward primer | GGCTAAGCAGAGCCTTGTTG |
| Reverse primer | TATTGCCGGGAAGTTCAGCA |
| **miR-21-5p** |  |
| Forward primer | TAGCTTATCAGACTGATGTTGA |
| Reverse primer | TGCGTGTCGTGGAGT |
| **SLC38A2** |  |
| Forward primer | ATGAGTTGCCTTTGGTGATCC |
| Reverse primer | ACAGGACACGGAACCTGAAAT |
| **GADPH** |  |
| Forward primer | CTGGGCTACACTGAGCACC |
| Reverse primer | AGTGGTCGTTGAGGGCAATG |
| **U6** |  |
| Forward primer | CTCGCTTCGGCAGCACA |
| Reverse primer | AACGCTTCACGAATTTGCGT |
